# Associations Between Socio-Economic Status and Child Health: Findings of a Large German Cohort Study

**DOI:** 10.3390/ijerph16050677

**Published:** 2019-02-26

**Authors:** Tanja Poulain, Mandy Vogel, Carolin Sobek, Anja Hilbert, Antje Körner, Wieland Kiess

**Affiliations:** 1 LIFE Leipzig Research Center for Civilization Diseases, Leipzig University, Philipp-Rosenthal-Strasse 27, 04103 Leipzig, Germany; mvogel@life.uni-leipzig.de (M.V.); csobek@life.uni-leipzig.de (C.S.); antje.koerner@medizin.uni-leipzig.de (A.K.); wieland.kiess@medizin.uni-leipzig.de (W.K.); 2 Department of Women and Child Health, Hospital for Children and Adolescents and Center for Paediatric Research (CPL), Leipzig University, Liebigstrasse 20a, 04103 Leipzig, Germany; 3 Integrated Research and Treatment Center AdiposityDiseases, Leipzig University, Philipp-Rosenthal-Strasse 27, 04103 Leipzig, Germany; anja.hilbert@medizin.uni-leipzig.de; 4 Department of Medical Psychology and Medical Sociology and Department of Psychosomatic Medicine and Psychotherapy, Leipzig University, Phillip-Rosenthal-Strasse 55, 04103 Leipzig, Germany

**Keywords:** socio-economic status, children, health, health behavior

## Abstract

The familial social background of a child can significantly impact their behavior and health. We investigated associations between socio-economic status (SES) and health parameters and behaviors in German children and adolescents. Data were collected between 2011 and 2018 in the framework of the LIFE Child study. Participants included 2998 children aged 3–18 years. SES was represented by an index combining information on parental education, occupation, and income. Associations between SES and health outcomes were estimated using linear and logistic regression analyses. In a moderator analysis, all associations were checked for interactions between SES and age or sex. A higher SES composite score was associated with better health (lower body mass index (*β* = −0.26), fewer behavioral difficulties (*β* = −0.18), higher quality of life (*β* = 0.21), fewer critical life events (odds ratio (OR) = 0.93); all *p* < 0.05) and a healthier lifestyle (healthier nutrition (*β* = 0.16), less excessive television use (OR = 0.87), less nicotine consumption (OR = 0.93), and more physical activity (OR = 1.18); all *p* < 0.05). However, SES was not associated with alcohol consumption (OR = 1.02) or sleep problems (*β* = −0.04). The strengths of the associations between SES and child health did not differ depending on SES indicator (education, occupation, income). The associations between SES and parent-reported behavioral difficulties and physical activity were stronger in older vs. younger children. In contrast, none of the observed associations were moderated by sex. This study highlights the strong association between socio-economic status and child health, even in modern Western societies.

## 1. Introduction

The social background of a family significantly impacts family residence, health care use or access, parenting, and, directly and indirectly, child behavior and development [1,2,3,4,5,6,7]. A central indicator of a family’s social background is the socio-economic status (SES), which is usually reflected by parents’ education, occupation, and (equivalent household) income [8].

Previous studies showed that children growing up in families with a lower SES have a higher body mass index (BMI) [9,10,11,12], report more behavioral problems [13,14,15], more psychosomatic symptoms [16], a lower quality of life [17,18], less healthy nutrition [19,20], less physical activity [16,21,22,23], a higher media consumption [22,23,24,25,26], and experience more critical life events than children growing up in families with a higher SES [27]. There is further evidence of associations between SES and laboratory parameters, e.g., serum lipid levels [28] and hemoglobin [29]. Some studies reported smaller associations between SES and health outcomes in adolescents compared to children or adults [30,31,32]. This decrease of health inequality during adolescence is explained by an increasing influence of school and peers relative to family and home [33]. With respect to the relative importance of the different SES indicators—education, occupation, and income—previous findings are mixed. Education and prestige- and income-related indicators of SES have been reported to show especially strong associations with different health parameters [11,20,34].

Despite the intensive research in the field of SES and child health, several research gaps exist. Most research projects only focused on a few health outcomes. Most studies investigated only one of the main SES indicators or used a composite score instead of comparing different SES indicators. The aim of the present study was to fill these research gaps by investigating associations of SES main indicators (education, occupation, and income) and a SES composite score with a wide range of health behaviors (nutrition, sleep, TV use, substance use, and physical activity) and health outcomes (BMI, behavioral difficulties, quality of life, and critical life events) in a large sample of German children and adolescents. Child age and sex were considered as factors that might moderate associations between SES and child health.

Based on previous study findings, we hypothesized that better health and a healthier lifestyle would be observed in children from higher social milieus. The strength of association was not expected to differ significantly depending on SES indicator. However, we expected to find evidence of a decrease in health inequality in adolescence.

## 2. Materials and Methods

### 2.1. Participants

The data for the present analyses were collected in the framework of the LIFE Child study (Leipzig, Germany), a longitudinal cohort study on the development of children [35,36]. The study was designed in accordance with the declaration of Helsinki and under the supervision of the Ethics Committee of the Medical Faculty of the University of Leipzig (Reg. No. 264/10-ek). Children are recruited until the age of 16 and invited to participate in yearly follow-up visits. For the present analyses, we only considered the last visit of each child.

The health outcomes and behaviors that were checked for associations with SES are listed in Table 1. The sample sizes differed depending on the specific measure, the age range (3–18 years, 3–10 years, or 10–18 years), and the acquisition period. The largest sample (BMI) consisted of 2998 3- to 18-year-old children. The smallest sample comprised 736 10- to 18-year-old children (self-reported nutrition).

### 2.2. Assessment of SES

The SES of children was assessed via a parental questionnaire originally designed for the use in the German Health Interview and Examination Survey for Children and Adolescents (KiGGS), a nation-wide survey on the health of children growing up in Germany [37]. In this questionnaire, parents provide information on their education (highest school degree and highest professional qualification), their occupational status (professional position), and the family net income. The family net income is transformed to the equivalent household income by accounting for the number of family members living in the same household. For each SES indicator, scores ranging between 1 and 7 can be assigned, with higher values indicating higher education, occupational status, and income.

Single scores can be summed into a composite score: the Winkler index. This index ranges between 3 and 21, with higher scores indicating higher SES. Based on the KiGGS guidelines [37], only the highest education and the highest occupational status of both parents are considered to calculate the SES index (in the cases where information was available for both parents). Missing information in a single SES indicator is replaced by the mean scores of the remaining two SES indicators. The index allows classification of families as lower (index ranging between 3 and 8.4), middle (index ranging between 8.5 and 15.4), or higher SES (index ranging between 15.5 and 21) [37]. In a representative sample, 20% of the sample should belong to the lower SES, 60% to the middle SES, and 20% to the higher SES [37].

### 2.3. Health Assessments

Table 1 shows the health behaviors and outcomes that were checked for associations with SES. From the age of 3 to 10 years, questionnaires were completed by parents (parent reports). From 10 to 18 years, questionnaires were completed by children and adolescents themselves (self-reports). A brief summary of the health behaviors and outcomes is provided in the following.

#### 2.3.1. Health Outcomes

Children’s height and weight were assessed by trained research assistants. The original BMIs were transformed into standard deviation scores (BMI SDS), based on German age- and sex-specific references [38].

Behavioral difficulties of children were assessed using the parent report and the self-report version of the Strengths and Difficulties Questionnaire (SDQ) [39]. For our analysis, the total difficulties score (sum score of all problem scales) was included in the analyses. This score ranges between 0 and 20, with higher scores indicating more behavioral difficulties.

Children’s quality of life was investigated by the self-report version of the KIDSCREEN-27 [40]. The sum score of all items (ranging between 5 and 135, with higher scores indicating higher quality of life) was included in the analyses.

Critical life events of adolescents were assessed using a self-created questionnaire assessing which of the following life events have been experienced in the last 6 months: violence, moving to another place, repetition of class, parents’ separation, death of relatives, and separation from friends. For further analysis, the experience of at least one critical life event was contrasted with the experience of none of these critical life events.

#### 2.3.2. Health Behaviors

The diets of children and adolescents were assessed using the instrument Composition and Culture of Eating (CoCu), a short questionnaire on the nutrition of children and adolescents [41]. Based on the given responses, a Nutritional Health Score can be derived. This score ranges between −120 (very unhealthy diet) to 120 (very healthy diet).

Sleep problems in children were assessed using the Child Sleep Health Questionnaire (CSHQ), a parent report on different sleep habits and problems of children [42]. The sum score of all subscales (ranging between 33 and 99, with higher scores indicating more sleep-related problems) was used for the present analyses. 

TV use was assessed by asking children (self-report) or their parents (parent report) how many hours per day the child usually spends in front of the TV. For further analyses, we investigated how many children met current recommendations on media consumption times, which was a maximum of 30 min per day for 3- to 10-year-old children (parent report) and maximum 1–2 h per day for older children (self-report) [43].

In a self-created questionnaire on substance use, adolescents were asked if they have ever smoked and if they have ever drank alcohol (response categories: yes or no). The responses to both questions were included in further analyses.

Physical activity was assessed by asking children (self-report) or their parents (parent report) how many times per week the child is physically active in sports clubs. For further analysis, we compared children who are physically active at least once vs. less frequently than once per week.

### 2.4. Statistical Analysis

All analyses were performed using R version 3.2.2 (R Foundation for Statistical Computing, Vienna, Austria) [44]. (Generalized) linear models were calculated to assess associations between SES and health outcomes or behaviors. We applied mixed-effect models that control for family relationships within the study sample. The package used for the calculation of the mixed-effect models was lme4 [45]. The *p*-values for each association were estimated using simultaneous tests as implemented in the multcomb package [46]. The significance level was set to *α* = 0.05. For all health outcomes or behaviors that were represented by continuous measures (BMI, behavioral difficulties, quality of life, nutrition, sleep problems), we applied linear mixed-effect models. For all health outcomes or behaviors represented by dichotomous measures (critical life events, TV use, smoking, alcohol consumption, physical activity), we applied generalized linear mixed-effect models. All associations were adjusted for age and sex.

For each health outcome or behavior, four different models were calculated. Each model contained the health outcome or behavior as dependent variable. In model 1 (the initial analysis) the SES composite score was considered as independent variable. In models 2 to 4 (the specific analysis), either maternal education, maternal occupational status, or income were included as the independent variable. We decided to include the education and occupational status of mothers as this information was more frequently available for mothers than for fathers.

For model 1, we considered all children for which the SES composite score was available. Therefore, missing information on one SES indicator might have been replaced by information on the other two indicators, following Lampert et al. [37]. For models 2 to 4, in contrast, only children whose parents had provided complete information on all three SES indicators were considered.

In a moderator analysis, all associations reported in the initial and specific analysis were checked for interactions with sex or age.

## 3. Results

### 3.1. SES Distribution

Table 2 summarizes the SES distribution according to the different SES indicators in the LIFE Child study. The classification to the lower (14%), middle (57%), or higher (29%) SES group demonstrates a slight under-representation of the lower SES group and an over-representation of the higher SES group in the present sample (compared to a representative German sample [37]). 

### 3.2. Associations Between SES Composite Score and Health Outcomes and Behaviors (Initial Analysis)

The associations between the SES composite score and the different health outcomes and behaviors (initial analysis) are displayed in Table 3. For BMI, behavioral difficulties, critical life events, TV use, and smoking, the analyses revealed significant negative associations with SES. For healthy nutrition and physical activity, we observed significant positive associations with SES. For sleep problems and alcohol consumption, no significant associations with SES were found.

For TV use and behavioral difficulties, the associations with SES were stronger if the behavior was assessed via the parent report vs. self-report version of the questionnaire. Regarding TV use, the confidence intervals of associations based on parent vs. self-report did not overlap (Table 3), indicating that the differences in the strengths of these associations were significant. For behavioral difficulties, the overlap between confidence intervals of the parent vs. self-report version was minimal (−0.31 to −0.22 vs. −0.23 to −0.13).

### 3.3. Associations Between Single SES Indicators and Health Outcomes and Behaviors (Specific Analysis)

Table 4 summarizes the associations of the single SES indicators (maternal education, maternal occupational status, and family income) with the health outcomes and behaviors (specific analysis). Overall, we observed that nearly all health outcomes or behaviors that showed significant associations with the SES composite score were also significantly associated with each of the single SES indicators. Only a few exceptions were observed: maternal education was not significantly associated with parent-reported physical activity. Maternal occupational status showed no significant association with critical life events. Family income was not significantly associated with healthy nutrition and smoking.

Taken together, maternal education and occupational status were significantly associated with 11 (out of 15) health outcomes and behaviors, and family income was significantly associated with 9 health outcomes. For each health outcome or behavior, the confidence intervals (CIs) of the associations with the single SES indicators overlapped strongly, indicating that the strengths of associations did not differ significantly as a function of SES indicator.

### 3.4. Child Age or Sex as a Moderator (Moderator Analysis)

The moderator analysis revealed several significant interactions between SES indicators and child age. In more detail, we found significant interactions between child age and the SES composite score for parent-reported behavioral difficulties (*β* = −0.12 (−0.18 to −0.06), *p* = 0.031) and parent-reported physical activity (OR = 1.04 (1.02–1.06), *p* < 0.001). Looking at the single SES indicators, significant interactions between child age and income were observed for BMI (*β* = −0.17 (−0.28 to −0.05), *p* < 0.001), parent-reported behavioral difficulties (*β* = −0.25 (−0.43 to −0.08), *p* = 0.013), and parent-reported physical activity (OR = 1.12 (1.03–1.22), *p* = 0.012). All interactions indicated stronger associations between SES and health outcomes or behaviors in older vs. younger children. However, these significant interactions between SES and child age were observed in parent-reported health behaviors or outcomes only, i.e., in the age group 3–10 years. For self-reported health outcomes, i.e., children aged 10–18 years, we found no significant interactions between SES and age.

In contrast to child age, sex did not moderate the associations between SES and child health.

## 4. Discussion

We comprehensively investigated associations between SES and several health outcomes and behaviors in a large sample of German children and adolescents. The strength of the study is that the three main SES indicators (education, occupation, and income) and a SES composite score were considered and contrasted. The moderating role of child age and sex was investigated.

### 4.1. Associations of SES with Child Health

This study showed that children with a lower SES have a higher BMI, have more behavioral difficulties, report a lower quality of life, and experience more critical life events than children with a higher SES. A lower SES was associated with a less healthy lifestyle (in terms of nutrition, smoking, TV use, and physical activity). Overall, the strengths of these associations did not differ significantly as a function of SES indicator (composite score, maternal education, maternal occupation, or income), suggesting that the impacts of the different SES indicators on child health are comparably strong.

The results of this study are in line with our hypotheses and the results reported by previous studies [9,10,11,12,13,14,15,16,17,18,19,21,22,23,24,25,47]. The findings suggest that social background is a determinant of child development and that children growing up in families with low SES might be at higher risk of developing diseases such as overweight or behavioral problems.

The consumption of alcohol was not associated with SES. This finding is in line with a systematic review showing no significant associations between SES and alcohol consumption [20]. However, the present study only assessed whether children have ever drank alcohol or not. Future studies might investigate whether type and frequency of alcohol consumption differ depending on social class. Similar to the consumption of alcohol, sleep-related problems were not associated with SES. This is in line with another study [48], which suggests that sleep-related problems in children (as well as factors that might explain variance in sleep, e.g., bedtime rituals, chronotype) are not affected by their family’s SES.

### 4.2. Associations of SES and Child Health Depending on Sex and Age

We found that the link between SES and child health is independent of child sex. However, our analyses suggest that child age might moderate the associations between SES and child health. Some associations between SES and child health (behavioral difficulties and physical activity) were stronger in older (e.g., 10-year-old) children compared to younger (e.g., 3-year-old) children. This finding indicates that the importance of SES increases and that the effects of the social milieu cumulate in this time period. For behavioral difficulties and TV use, associations appeared to be stronger in younger (3- to 10-year-old children) compared to older (10- to 18-year-old) children. These findings suggest that the influence of SES or SES-related factors on behavioral difficulties and media use is stronger during childhood than during adolescence. A decrease in health inequality during adolescence has been reported in several studies [30,31,32]. In this period of development, peers and school environment may play more important roles than family and social background, which, consequently, might diminish the influence of the family’s SES [33]. However, the associations between SES and health were highly significant even during adolescence, indicating that the effect of the social background may diminish but not disappear during adolescence.

Notably, the differences in associations observed in younger (3- to 10-year-old) vs. older (10- to 18-year-old) children might be partly explained by differences in the acquisition method. In younger children, the questions on children’s health were answered by parents. Older children or adolescents, in contrast, completed questionnaires on their own. We cannot rule out that parents with a higher SES show more social desirability (and therefore report healthier behavior and better health in their children) than parents with a lower SES.

### 4.3. Implications

The findings of this study indicate that the impact of social background on behavior and health starts early in development. They underline the importance of strengthening the awareness of health inequality in pediatric care systems and in politics. Education is an SES indicator that develops early and might be most effectively controlled. Therefore, more efforts are needed to ensure early and ongoing quality education independently of a child’s social background, e.g., by introducing free (but obligatory) whole-day-offerings for children. Regular exercise and programs on healthy behavior and the potential risks of unhealthy behavior should be integral parts in the curriculums of kindergartens and schools.

### 4.4. Limitations and Future Directions

The main limitation is the distribution of SES in the present study. Whereas the middle social milieu and the high social milieu were well represented, the low social milieu was under-represented. The uneven distribution of social classes might limit the generalizability of our study findings to the general population. The SES information used in the initial analysis differed slightly from the information used in the specific analysis. Whereas the initial analyses included information on mothers and fathers (if available), the specific analysis did not. Therefore, the results of the different analyses lack a certain comparability. Finally, although this study assessed associations between SES and a large amount of health outcomes, the assessments themselves reflect rather rough measures of child health.

Future studies might investigate whether maternal and parental education, occupation, and income show the same associations with child health. The role of SES in specific facets of child health might be investigated in more detail (e.g., associations between SES and single domains of quality of life, sleep problems, or nutrition). A more detailed analysis might show which factors, e.g., aspects of child or parental behavior and environment, mediate the association between SES and health outcomes. Based on the findings, programs and political initiatives aiming at minimizing social inequality might be developed, evaluated, and implemented.

## 5. Conclusions

This study showed that children growing up in families with a higher SES are in better health and adopt a healthier lifestyle than children growing up in families with a lower SES. The analyses suggest that associations of SES with health increase during early childhood but diminish in older childhood and adolescence.

## Figures and Tables

**Table 1:** Description of study samples and assessments.

	Measure	Assessment	Age (Years)	Acquisition Years	*N*
**Health outcome**	BMI	E	3–18	2011–2018	2998
	Behavioral difficulties	Q (SR)	10–18	2011–2018	1547
		Q (PR)	3–10	2011–2018	2007
	Quality of life	Q (SR)	10–18	2011–2018	1636
	Critical life events	Q (SR)	10–18	2011–2017	1559
**Health behavior**	Nutrition	Q (SR)	10–18	2016–2018	736
		Q (PR)	3–10	2016–2018	868
	Sleep problems	Q (PR)	3–10	2011–2015	943
	TV use	Q (SR)	10–18	2011–2017	1486
		Q (PR)	3–10	2011–2017	1828
	Smoking	Q (SR)	10–18	2011–2017	1350
	Alcohol consumption	Q (SR)	10–18	2011–2017	1350
	Physical activity	Q (SR)	10–18	2011–2017	1488
		Q (PR)	3–10	2011–2017	1828

BMI: body mass index, E: Examination, Q: Questionnaire, PR: parent report, SR: self-report.

**Table 2:** Socio-economic status (SES) in the LIFE Child study.

SES Indicator	*N* Families	Range	Mean (SD)	Classification
SES composite score	2590	3–21	12.93	14% low, 57% middle, 29% high
Education mother	2164	1–7	3.49	
Occupational status mother	2281	1–7	4.75	
Household equivalent income	2548	1–7	4.33	

**Table 3:** Associations of SES (as indicated by the SES composite score) with health outcomes and behaviors in children.

**Continuous Outcomes**	**Age (Years)**	***N***	***β*** **(95% CI)**	***p***
BMI	3–18	2998	−0.26 (−0.30 to −0.22)	<0.001
Behavioral difficulties (SR)	10–18	1547	−0.18 (−0.23 to −0.13)	<0.001
Behavioral difficulties (PR)	3–10	2007	−0.27 (−0.31 to −0.22) *	<0.001
Quality of life (SR)	10–18	1636	0.21 (0.16–0.26)	<0.001
Score healthy nutrition (SR)	10–18	736	0.16 (0.09–0.24)	<0.001
Score healthy nutrition (PR)	3–10	868	0.15 (0.08–0.23)	<0.001
Sleep problems (PR)	3–10	943	−0.04 (−0.11 to 0.03)	0.620
**Binary Outcomes**	**Age Range**	***N***	**OR (95% CI)**	***p***
Critical life events (SR)	10–18	1559	0.93 (0.90–0.96)	<0.001
TV use (SR)	10–18	1486	0.87 (0.87–0.88)	<0.001
TV use (PR)	3–10	1828	0.75 (0.68–0.82)	<0.001
Smoking (SR)	10–18	1350	0.93 (0.89–0.98)	0.002
Alcohol consumption (SR)	10–18	1350	1.02 (0.98–1.06)	0.405
Physical activity (SR)	10–18	1488	1.18 (1.13–1.22)	<0.001
Physical activity (PR)	3–10	1828	1.16 (1.09–1.24) *	<0.001

All associations are adjusted for age and sex. BMI: body mass index, SR: self-report, PR: parent report; CI: confidence interval, OR: odds ratio. * Significant interactions with child age indicate stronger associations in older vs. younger children.

**Table 4:** Associations of different health outcomes and behaviors with children’s SES (as indicated by maternal education, maternal occupational status, or equivalent household income).

Continuous Outcomes	Age (Years)	*N*	Maternal Education		Maternal Occupational Status		Equivalent Household Income	
			*β* (95% CI)	*p*	*β* (95% CI)	*p*	*β* (95% CI)	*p*
BMI	3–18	2226	−0.20 (−0.25 to −0.15)	<0.001	−0.19 (−0.23 to −0.14)	<0.001	−0.15 (−0.20 to −0.11) *	<0.001
Behavioral difficulties (SR)	10–18	1150	−0.12 (−0.18 to −0.06)	<0.001	−0.12 (−0.17 to −0.06)	<0.001	−0.14 (−0.20 to −0.08)	<0.001
Behavioral difficulties (PR)	3–10	1525	−0.17 (−0.22 to −0.12)	<0.001	−0.18 (−0.23 to −0.13)	<0.001	−0.15 (−0.20 to −0.10) *	<0.001
Quality of life (SR)	10–18	1210	0.12 (0.07–0.18)	<0.001	0.14 (0.08–0.20)	<0.001	0.16 (0.11–0.22)	<0.001
Score healthy nutrition (SR)	10–18	570	0.15 (0.07–0.24)	<0.001	0.14 (0.05–0.22)	0.004	0.10 (0.01–0.18)	0.069
Score healthy nutrition (PR)	3–10	653	0.14 (0.05–0.22)	0.005	0.12 (0.04–0.20)	0.018	0.05 (−0.03 to 0.13)	0.531
Sleep problems (PR)	3–10	719	−0.03 (−0.11 to 0.05)	0.817	−0.03 (−0.10 to 0.05)	0.877	0.03 (−0.05 to 0.10)	0.883
**Binary Outcomes**			**OR (95% CI)**	***p***	**OR (95% CI)**	***p***	**OR (95% CI)**	***p***
Critical life events (SR)	10–18	1157	0.87 (0.78–0.96)	0.005	0.88 (0.76–1.02)	0.095	0.70 (0.57–0.86)	<0.001
TV use (SR)	10–18	1107	0.75 (0.66–0.85)	<0.001	0.73 (0.60–0.88)	0.001	0.52 (0.39–0.70)	<0.001
TV use (PR)	3–10	1384	0.60 (0.51–0.69)	<0.001	0.49 (0.39–0.62)	<0.001	0.55 (0.42–0.71)	<0.001
Smoking (SR)	10–18	992	0.86 (0.74–0.99)	0.044	0.61 (0.47–0.79)	<0.001	0.77 (0.58–1.02)	0.069
Alcohol consumption (SR)	10–18	992	1.04 (0.91–1.18)	0.540	1.01 (0.83–1.23)	0.907	0.83 (0.64–1.07)	0.149
Physical activity (SR)	10–18	1108	1.33 (1.18–1.50)	<0.001	1.65 (1.38–1.98)	<0.001	1.83 (1.45–2.31)	<0.001
Physical activity (PR)	3–10	1384	1.11 (0.99–1.25)	0.068	1.57 (1.30–1.91)	<0.001	1.42 (1.14–1.77) *	0.002

All associations are adjusted for age and sex. BMI: body mass index, SR: self-report, PR: parent report. * Significant interactions with child age indicate stronger associations in older vs. younger children.
